# Safety and efficacy of *two-step peginterferon α-2a treatment* in patients of chronic hepatitis B with acute exacerbation

**DOI:** 10.1111/j.1365-2893.2011.01469.x

**Published:** 2012-03

**Authors:** C-C Chen, P-C Wang, H-W Chang, C-F Chen

**Affiliations:** Division of Hepatology & Gastroenterology, Department of Internal Medicine, Mackay Memorial HospitalHsin-Chu, Taiwan

**Keywords:** chronic hepatitis B, entecavir, HBeAg seroconversion, HBsAg clearance, Pegasys, peginterferon α-2a

## Abstract

The focus of this study was to evaluate the safety and efficacy of sequential peginterferon α-2a (Pegasys) therapy for chronic hepatitis B with acute exacerbation [ALT > 10 × upper limit of normal (ULN), bilirubin <2.0 mg/dL]. Four groups of patients categorized by HBeAg status and treatment regimens were studied since May 2007. Nineteen HBeAg-positive patients (Group 1) had received entecavir  pretreatment  (when ALT > 10 × ULN) plus Pegasys (180 μg/kg/week, when ALT was 5–10 × ULN) for 24 weeks. Thirteen HBeAg-negative patients (Group 2) had the same protocol for 48 weeks. In both groups, entecavir was then discontinued 14 days after the initiation of Pegasys. The results were compared, respectively, to 35 HBeAg-positive patients (Group 3) and 24 HBeAg-negative patients (Group 4), all with ALT > 5 × ULN, under continual entecavir monotherapy. The ALT levels of patients in Group 1 and 2 who had received entecavir pretreatment for a duration of 19.63 ± 3.34 days were below four times of ULN following 4 weeks of Pegasys treatment. At week 96, the rates of sustained virological response were 69.2% (9/13) and 80% (8/10), and the relapse rates were 23.1% (3/13) and 11.2% (1/9) for HBeAg-positive and HBeAg-negative patients with *two-step Pegasys treatment*, respectively. The HBeAg seroconversion rates were 46.2% in Group 1, and 42.1% in Group 3; HBsAg loss rates were 15.4% (2/13) in Group 1, and 30% (3/10) in Group 2, whereas none achieved HBsAg loss with entecavir monotherapy (Group 3 and 4). The *two-step Pegasys treatment* offers an alternative, other than the nucleos(t)ides, for treating chronic hepatitis B with acute exacerbation and provides a safe, efficacious, short-term and finite strategy.

## Introduction

Two different treatment strategies are applicable in both HBeAg-positive and HBeAg-negative patients with chronic hepatitis B: Treatment of definite duration with pegylated interferon-α (PEG-INF) and long-term treatment with nucleoside/nucleotide analogues (NA). The 2009 EASL guidelines stated that ‘in HBeAg-positive and HBeAg-negative patients, the ideal end point of therapy is sustained HBsAg loss with or without seroconversion to anti-HBs [1].’ However, it is not a practical goal for most patients and has not been achieved by the majority of patients thus far. In HBeAg-positive patients, durable HBe seroconversion is a satisfactory end point because it has been shown to be associated with improved prognosis [1,2]. The next most desirable end point in HBeAg-positive patients who do not achieve HBe seroconversion, and in HBeAg-negative patients, is a sustained undetectable HBV DNA level after interferon therapy or a maintained undetectable HBV DNA level on treatment with NA [1]. The most potent NA can suppress HBV DNA levels to undetectable levels up to 80–90% of patients. Among them, only a few sustain the response after 6 months of treatment discontinuation [3,4]. Because to achieve a post-therapy sustained response seems more difficult in HBeAg-negative patients [5], indefinite NA treatment is often required and eventually leads to emergence of resistant mutants and side effects. Hence, peginterferon α-2a is the choice for HBeAg-negative patients [6].

Patients are most likely to benefit from interferon therapy if high ALT levels (>3 × ULN, upper limit of normal), low HBV DNA (various definitions ranging from <2 × 10^6^ to 2 × 10^8^ IU/mL) detected [1,7]. In addition, HBV genotypes A and B have been associated with a better response to interferon than genotypes C and D [8–11]. However, the HBV genotype has been suggested as a poor individual predictive value and should not be the role factor to determine types of treatment initiated [1]. Patient characteristics associated with favourable peginterferon therapy outcomes include younger age [12,13], lack of comorbidities and high motivation to adhere to treatment. The main advantages of peginterferon are as follows: absence of resistance, finite duration of therapy, possibility for immune-mediated clearance of HBV (HBsAg loss), higher HBeAg seroconversion in the first year of therapy, higher durability and off-treatment (delay) response [1]. Owing to its finite duration of therapy, it is also the choice for nonpregnant women of child-bearing age. The disadvantages of peginterferon therapy include the following: frequent adverse effects, poor tolerance, moderate antiviral effect, the necessity for subcutaneous injections and close monitoring.

In practice, the interferon is relative contraindication for chronic hepatitis B with acute exacerbation (ALT levels >10 × ULN), considering its side effects and at time may induce flare up and decompensation of liver function, as indicated in the 2000 APASL guidelines that ‘For HBV DNA seropositive patients with an ALT level >5 × ULN, lamivudine is recommended because of its efficacy, and rapidity of action, whereas interferon carries the potential to precipitate decompensation [14].’ Nonetheless, when ALT levels are reduced to 5–10 times ULN, patients can be re-evaluated and prescribed the suitable protocols that were discouraged during the initial stage. We developed a new protocol to apply NA and PEG-INF in a sequential scheme that showed PEG-INF is applicable in patients of chronic hepatitis B with acute exacerbation. We assessed the results by comparing the outcomes of patients undergoing these two different strategies and found the ‘*two-step peginterferon α-2a treatment*’ to be of safety and efficacy in this study.

## Patients and Methods

### Patients

All patients (25.6–43.5 years old) were interferon naïve and had positive HBsAg (>250 IU/mL) for at least 12 months, serum HBV DNA of at least 500 000 copies/mL, and at least two episodes of increasing ALT levels of 1.5–10 times ULN within 12 months before enrolment. Patients with liver decompensation, advanced fibrosis, cirrhosis and hepatoma were excluded. Other cause of chronic liver disease should be systematically checked to exclude co-infection with HDV, HCV and HIV, comorbidities with alcoholism, autoimmune and metabolic liver disease. Serious medical or psychiatric illnesses that had usage of corticosteroid or immunosuppressive agents at the time of study were excluded. All patients in this study lived in Hsinchu (Taiwan), a city of 400 000 populations, with same demographics. Owing to patients’ fear or refusal of liver biopsy, three patients (3/32) had the liver biopsy and the rest relied on other clinical methods to obtain equivalent information of patient conditions. In our cases, the higher necroinflammation scores were derived by the ALT and HBV DNA level; ultrasonorgraphy helped to filter out patients with advanced fibrosis. The liver sonar examination was performed by two experienced hepatologists at least three times on each patient.

### Study design and follow-up

Before treatment, the patient were counselled on the advantages and disadvantages of taking peginterferon *vs* nucleos(t)ide analogue, and the treatment regimens were decided by themselves. This was a prospective, nonrandomized, open-label study that evaluated the safety and efficacy of sequential peginterferon α-2a (Pegasys) therapy for chronic hepatitis B patients with acute exacerbation, when ALT levels were above 10 times ULN (>400 IU/L) and total bilirubin were below 2.0 mg/dL. All patients provided informed written consent.

**Fig. 1:**
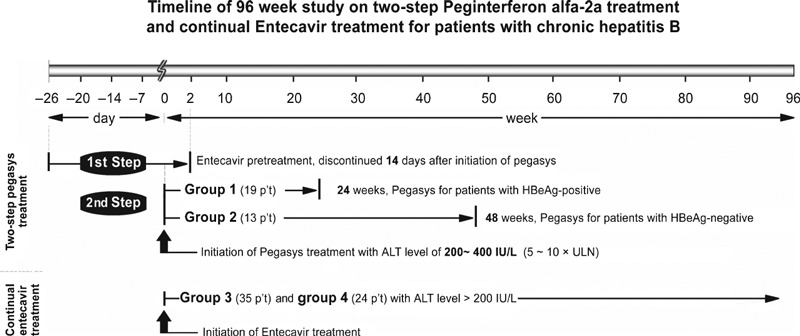
Timeline of 96 week study on two-step peginterferon alfa-2a treatment and continual entecavir treatment for patients with chronic hepatitis B.

A new strategy was developed to decrease HBV DNA viral loads and ALT levels firstly when ALT levels were over 10 times ULN by induction treatment with entecavir (0.5 mg) before the initiation of peginterferon α-2a (Pegasys). Entecavir was prescribed as pretreatment when ALT levels >10 × ULN and was discontinued 14 days after initiation of Pegasys upon the decline of ALT to 5–10 × ULN (200–400 IU/L). This sequential strategy is called *‘two-step Pegasys treatment’* for chronic hepatitis B with acute exacerbation. Since May 2007, there were four groups of patients categorized by HBeAg status and treatment regimens. Nineteen HBeAg-positive patients (Group 1) had received entecavir (0.5 mg daily) pretreatment plus Pegasys (180 μg/kg/week) subcutaneously for 24 weeks. Thirteen HBeAg-negative patients (Group 2) had the same protocol for 48 weeks. Pegasys was added when ALT levels had decreased to 5–10 times ULN in patients of Group 1 and Group 2 during entecavir pretreatment. In short, the first step is pretreatment with entecavir when ALT >400 IU/L; the secondary step is to add Pegasys when ALT levels decline to 200–400 IU/L and still maintain entecavir for another 14 days (Fig. 1).

For comparison, we also enrolled patients with entecavir monotherapy for ALT levels above five times ULN. Thirty-five HBeAg-positive patients (Group 3) and 24 HBeAg-negative patients (Group 4) were prescribed with entecavir (0.5 mg daily) alone for ALT levels >200 IU/L and were kept on treatment during the 96 weeks of study. The ALT levels were divided into >400 and 200–400 IU/L in Group 3, and the rates of HBeAg seroconversion were analysed, respectively.

ALT levels were monitored weekly during the first 4 weeks of starting Pegasys therapy and every 2 weeks afterwards during the first 5–12 weeks. HBV DNA levels were measured before introducing entecavir and Pegasys, and on the 12th, 24th, 48th, 72nd and 96th week after adding Pegasys. The same schedules of measurement were applied to those received entecavir monotherapy. Patient receiving Pegasys had blood counts, and ALT monitored every 2–4 weeks, thyroid stimulating hormone (TSH) and free T4 tests every 12 weeks during treatment. HBeAg/anti-HBe was tested every 12 weeks for HBeAg-positive patients, and HBsAg every 6 months.

### Laboratory tests

Serum HBV DNA was followed up by real-time PCR (polymerase chain reaction); the detection range was from 10^2^ to 10^9^ copies/mL. The undetectable HBV DNA level was defined as <100 copies/mL. All patients were tested for HBV genotyped by sequencing-based assays and for the precore G1896A and basal core promoter A1762T/G1764A mutants by direct sequencing. Quantitative HBsAg (qHBsAg) was quantified using Architect HBsAg QT (Abbot Diagnostic Division, Sligo, Ireland). The sensitivity of the Architect assay ranged from 0.05 to 250 IU/mL. All of our patients presented HBsAg titre >250 IU/mL before treatment, which was associated with a higher prevalence of chronic hepatitis. The HBsAg loss/clearance was defined as HBsAg titre <0.05 IU/mL. The HBeAg seroconversion was defined as of HBeAg loss and anti-HBe detection on two occasions 1–3 months apart; the ALT normalization, as ALT levels of ≦40 IU/L.

### Statistical analysis

Statistical analysis was performed using SPSS software version 18.0 (SPSS, Somers, NY, USA) and GraphPad InStat (GraphPad, La Jolla, CA, USA). ORIGINpro version 7.0 (OriginLab, Northampton, MA, USA) was used for linear fits and graphic plotting. Continuous variables are expressed as the (mean ± standard deviation) or median (IQR). Group comparison was performed using the Student’s t test, chi-square test or Fisher’s exact test as appropriate. Statistical significance was taken as a two-sided *P* value of <0.05.

## Results

The clinical characteristics and outcomes of four controlled groups are presented in Tables 1, 2 & S1. The primary objective in this study was the safety of *two-step Pegasys treatment*; the secondary issue was the efficacy and durability, including HBeAg seroconversion, HBsAg clearance and HBV DNA levels at week 48, 72 and 96.

**Table 1:** Clinical characteristics of HBeAg-positive chronic hepatitis B patients on 24-week *two-step peginterferon alfa-2a treatment* and continual Entecavir treatment for 96-week study

	Two-step Pegasys Treatment (Group 1)	Continual Entecavir Treatment (Group 3)	*P*-value
Baseline			
No. of patients	19	35	
Age (years)	33.38 ± 5.51 (25.6–43.2)†	33.25 ± 5.43 (24.5–42.5)†	0.9294
Male sex, *n* (%)	13 (68.4)	24 (68.6)	0.991
HBeAg positive, *n* (%)	19 (100)	35 (100)	
Genotype B: C	12:7		
Duration of Entecavir pretreatment (days)	19.32 ± 3.46 (12–26)†		
2-Step Pegasys treatment			
1st step (begin Entecavir) – ALT (IU/L)	723 (596–854)		0.1711 *
2nd step (begin Pegasys) – ALT (IU/L)	298 (230–359)		0.0167 **
Continual entecavir treatment (begin) – ALT (IU/L)		415 (279–749)	
2-Step Pegasys treatment			
1st step (begin) – HBV DNA (log cps/ml)	7.73 ± 1.11		0.1231 *
2nd step (begin) – HBV DNA (log cps/ml)	6.73 ± 1.02		0.0472 *
Continual entecavir treatment (begin)-HBV DNA (log cps/ml)		7.29 ± 0.93	
Results			
HBV DNA (log copies/ml)			
Week 12	4.45 ± 1.00	4.12 ± 0.70	0.1548
Week 24 (end of Pegasys treatment – 2nd step)	3.00 ± 1.04	2.67 ± 0.63	0.1537
Week 48	3.52 ± 1.34	2.11 ± 0.23	<0.0001
Week 72	3.58 ± 1.19	2.05 ± 0.14	<0.0001
Week 96	3.28 ± 1.16	2.04 ± 0.12	<0.0001
HBeAg seroconversion, *n*/total (%)‡			
Week 12	0/19 (0.0)	0/35 (0.0)	
Week 24 (end of Pegasys treatment – 2nd step)	8/19 (42.1)	2/35 (5.7)	0.001
Week 48	8/19 (42.1)	9/35 (25.7)	0.216
Week 72	8/18 (44.4)	11/26 (42.3)	0.888
Week 96	6/13 (46.2)	8/19 (42.1)	0.821
HBsAg loss, *n*/total (%)‡			
Week 12	0/19 (0.0)	none	
Week 24 (end of Pegasys treatment – 2nd step)	2/19 (10.5)		
Week 48	2/19 (10.5)		
Week 72	2/18 (11.1)		
Week 96	2/13 (15.4)	0/19 (0.0)	0.077
HBsAg seroconversion, *n*/total (%)‡			
Week 12	0/19 (0.0)	none	
Week 24 (end of Pegasys treatment – 2nd step)	1/19 (5.3)		
Week 48	1/19 (5.3)		
Week 72	1/18 (5.6)		
Week 96	1/13 (7.7)	0/19 (0.0)	0.219

Continuous values are expressed as the ‘mean ± standard deviation’ or ‘median (IQR)’. IQR denotes interquartile range.

† mean ± standard deviation (min-max).

‡ *n*/total: (patient with specified response/total number of patient) at specified week.

* Measures at the ‘begin of Entecavir (1st step)’*vs*‘begin of continual Entecavir’.

** Measures at the ‘begin of Pegasys (2nd step)’*vs*‘begin of continual Entecavir’.

**Table 2:** Clinical characteristics of HBeAg-negative chronic hepatitis B patients on 48-week *two-step peginterferon alfa-2a* treatment and continual Entecavir treatment for 96-week study

	Two-step Pegasys treatment (Group 2)	Continual entecavir treatment (Group 4)	*P-*value
Baseline			
No. of patients	13	24	
Age (years)	37.34 ± 3.20 (33.2–43.5)†	38.62 ± 4.54 (29.6–48.6)†	0.3738
Male sex, *n* (%)	10 (76.9)	17 (70.8)	0.69
HBeAg negative, *n* (%)	13 (100)	24 (100)	
Genotype B: C	11:2		
Duration of entecavir pretreatment (days)	20.08 ± 3.23 (16–26)†		
2-Step Pegasys treatment			
1st step (begin Entecavir) – ALT (IU/L)	612 (459–676.5)		0.4227 *
2nd step (begin Pegasys) – ALT (IU/L)	312 (277–373.5)		0.0399 **
Continual Entecavir treatment (begin) – ALT (IU/L)		378.50 (285.3–752.5)	
2-Step Pegasys treatment			
1st step (begin) – HBV DNA (log cps/ml)	6.68 ± 0.75		0.8503 *
2nd step (begin) – HBV DNA (log cps/ml)	5.81 ± 0.85		0.0189 **
Continual entecavir treatment (begin)-HBV DNA (log cps/ml)		6.62 ± 1.00	
Results			
HBV DNA (log copies/ml)			
Week 12	4.10 ± 0.64	3.92 ± 0.93	0.5568
Week 24	3.10 ± 0.66	3.02 ± 0.96	0.7836
Week 48 (end of Pegasys treatment – 2nd step)	2.89 ± 0.83	2.21 ± 0.53	0.0046
Week 72	3.72 ± 1.14	2.10 ± 0.28	<0.0001
Week 96	3.06 ± 1.04	2.03 ± 0.10	0.0025
HBsAg loss, *n*/total (%)‡			
Week 12	0/13 (0.0)	none	
Week 24	1/13 (7.7)		
Week 48 (end of Pegasys treatment – 2nd step)	3/13 (23.1)		
Week 72	3/13 (23.1)		
Week 96	3/10 (30.0)	0/12 (0.0)	0.041
HBsAg seroconversion, *n*/total (%)‡			
Week 12	0/13 (0.0)	none	
Week 24	1/13 (7.7)		
Week 48 (end of Pegasys treatment – 2nd step)	2/13 (15.4)		
Week 72	2/13 (15.4)		
Week 96	2/10 (20.0)	0/12 (0.0)	0.104

Continuous values are expressed as the ‘mean ± standard deviation’ or ‘median (IQR)’. IQR denotes interquartile range.

† Mean ± standard deviation (min-max).

‡ *n*/total: (patient with specified response/total number of patient) at specified week.

* Measures at the ‘begin of Entecavir (1st step)’*vs*‘begin of continual Entecavir’.

** Measures at the ‘begin of Pegasys (2nd step)’*vs*‘begin of continual Entecavir’

### Safety and adverse effects

Thirty patients (30/32, 94%) experienced more than one adverse effect in Pegasys-group (Group 1 & 2), with no unexpected or serious adverse events reported. The side effects of Pegasys were tolerable without early termination and reduction of the dose given. Only one HBeAg-positive female patient had hyperthyroidism at week 24, and Pegasys was just terminated upon the treatment completion. There was no severe hepatitis flare and decompensation reported for *two-step Pegasys treatment.* Readings of ALT remained below four times ULN after 4 weeks of Pegasys treatment, except one HBeAg-positive male patient had higher ALT levels (180–220 IU/L) at week 16–24. However, his HBV DNA level was only 425 IU/L, and the ALT level dropped below two times ULN 2 weeks into off-treatment later. The immune- or drug-mediated hepatitis was suspected the cause.

### HBeAg seroconversion

**Fig. 2:**
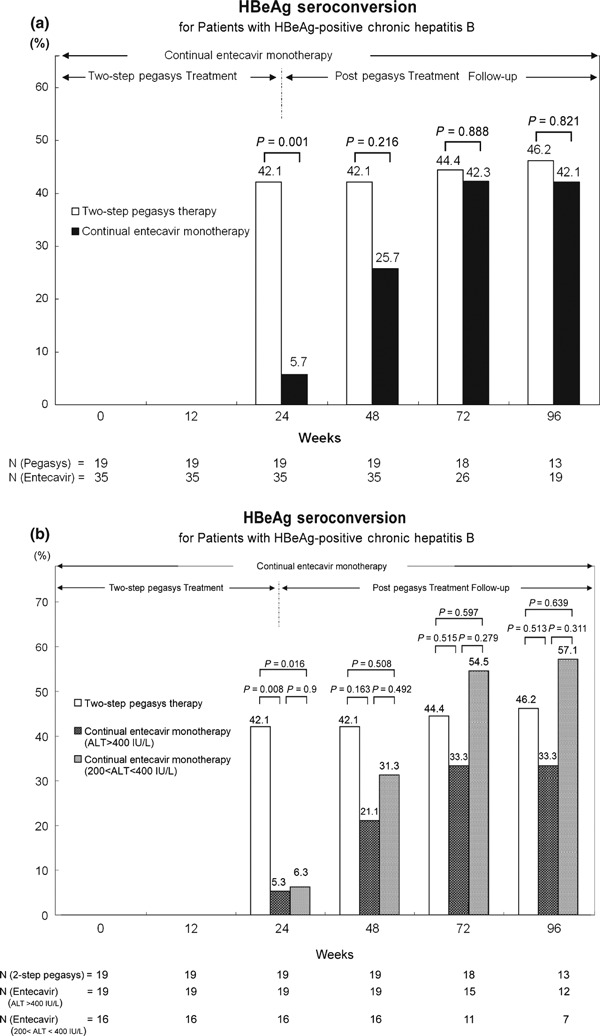
(a) The percentages of HBeAg seroconversion response after a 24-week two-step Pegasys treatment compared to the continual Entecavir monotherapy for patients with HBeAg-positive chronic hepatitis B. (Group 1 *vs* Group 3). (b) The percentages of HBeAg seroconversion for patients with HBeAg-positive chronic hepatitis B after a 24-week two-step Pegasys treatment compared with that of the continual Entecavir monotherapy for patients with ALT >400 and 200–400 IU/L before treatment (Group 1 *vs* G3 with >400 *vs* G3 with 200–400 IU/L)

Results of HBeAg-positive chronic hepatitis B patients shown in Table 1 indicate a continuous percentage growth of HBeAg seroconversion from 42.1% at the end of *two-step Pegasys treatmen* (24 weeks, Group 1) to 46.2% at week 96. One patient achieved HBeAg loss during follow-up at week 48. The rate of HBeAg seroconversion for patients under continual entecavir monotherapy (Group 3) rose from 5.7 to 42.1% at week 24 and 96, respectively (Fig. 2a). The patients in Group 3 were divided into ALT >400 and 200–400 IU/L, for comparison with the *two-step Pegasys treatment* (Group 1). The HBeAg seroconversion rates in patients treated by the *two-step Pegasys treatment*, continual entecavir treatment with ALT >400 and 200–400 IU/L at week 96 were 46.2, 33.3 and 57.1%, respectively (Fig. 2b). Patients in Group 1 (*two-step Pegasys treatment*) had higher rates of HBeAg seroconversion in the first year; however, no significant difference was observed at week 48, 72 and 96, except at week 24 (end of treatment in Group 1), as opposed to patients in Group 3 (*P* value = 0.008 for ALT > 400 IU/L and *P* value = 0.016 for 200–400 IU/L).

### HBsAg clearance

**Fig. 3:**
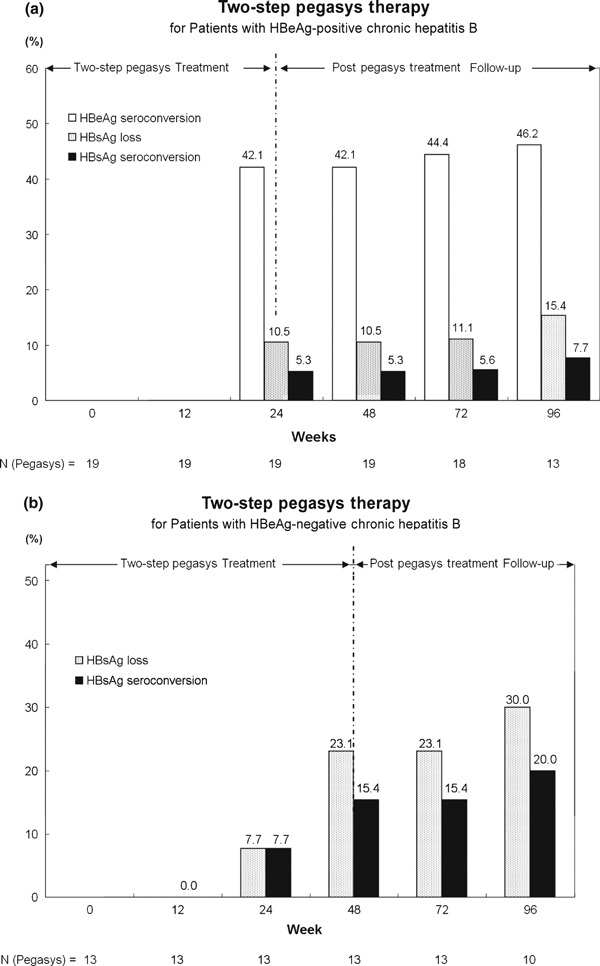
(a) Percentages of HBeAg-positive patients achieving HBeAg seroconversion, HBsAg loss and HBsAg seroconversion after a 24-week two-step Pegasys treatment. (Group 1). (b) Percentages of HBeAg-negative patients achieving HBsAg loss and HBsAg seroconversion after a 48-week two-step Pegasys treatment. (Group 2)

At week 96, HBsAg loss reached 15.4% (2/13) and HBsAg seroconversion (on subset of HBsAg loss) was 7.7% (1/13) in HBeAg-positive patients under 24 weeks *two-step Pegasys treatment* (among the whole patients in Group 1, Fig. 3a); whereas none for those who received entecavir (Table 1). Patients with HBeAg-negative chronic hepatitis B showed a ratio of 30% (3/10) HBsAg loss and 20% (2/10) of HBsAg seroconversion after 48 weeks of *two-step Pegasys treatment* (among the whole patients in Group 2, Fig. 3b), *vs* none produced by entecavir treatment at the end of study (week 96, Table 2). According to our records of further follow-up, such outcome sustained at week 120.

There were 32 patients enrolled in the *two-step Pegasys treatment*; 23 and nine patients were genotype B and C, respectively. Five patients with HBsAg loss were all genotype B (5/23), and three in the rest (18 patients) of genotype B without HBsAg loss had low titre of HBsAg (<5 IU/mL) and low HBV DNA levels (<1000 copies/mL) with ALT normalization at week 96. Based on the outcomes, genotype B seemed to have a trend to achieve HBsAg clearance. However, there was no significant difference (*P* value = 0.128) between genotype B and C possibly because of the small sample size and short-term follow-up.

### HBV DNA levels

**Fig. 4:**
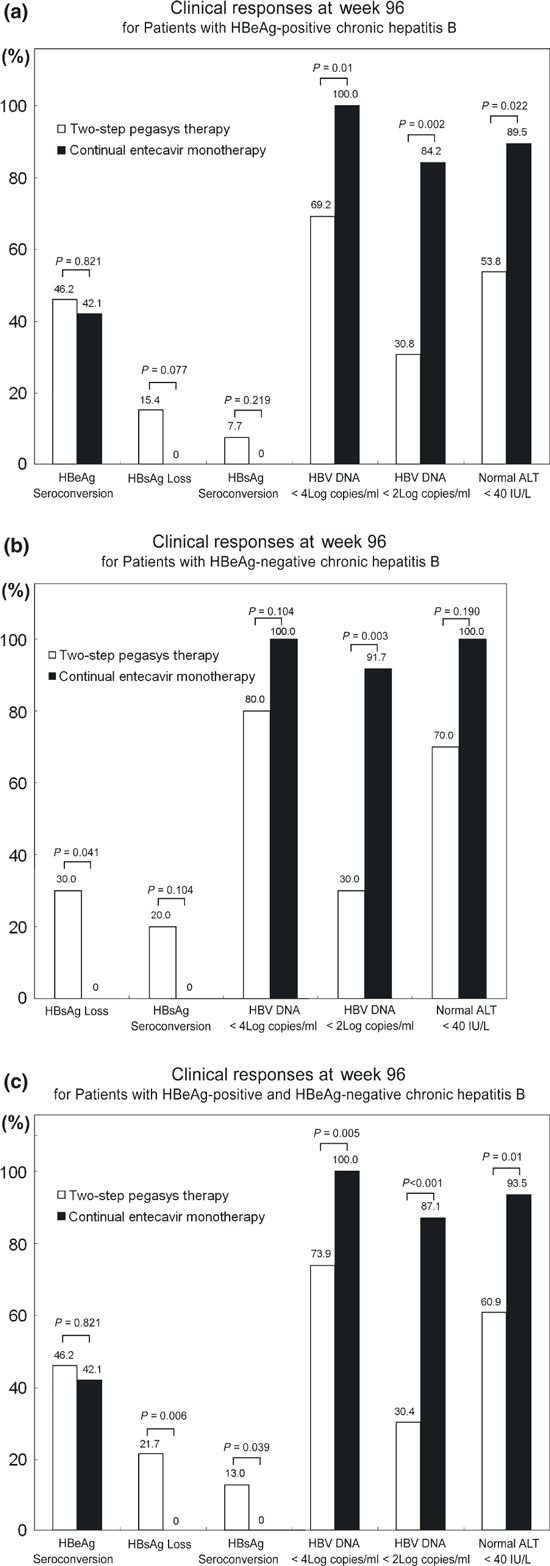
Clinical responses at week-96. (a) in patients with HBeAg-positive chronic hepatitis B received a 24-week two-step Pegasys treatment *vs* continual Entecavir treatment. G1 (*N* = 13) *vs* G3 (*N* = 19). (b) in patients with HBeAg-negative chronic hepatitis B received a 48-week two-step Pegasys treatment *vs* continual Entecavir treatment. G2 (*N* = 10) *vs* G4 (*N* = 12). (c) in patients with HBeAg-positive and HBeAg-negative chronic hepatitis B who had received a 24- and 48-week two-step Pegasys treatment respectively *vs* those under continual Entecavir treatment. G1 + 2 (*N* = 23) *vs* G3 + 4 (*N* = 31).

There is no primary nonresponse on the *two-step Pegasys treatment* or entecavir (defined as failure to achieve a log reduction of HBV DNA from baseline at week 12). Furthermore, all patients reached at least 3 log of HBV DNA levels reductions at week 12. Therefore, all patients maintained Pegasys treatment until week 24 and 48 for HBeAg-positive and HBeAg-negative, respectively. Sustained virological response (SVR)/durability for Pegasys treatment is defined as HBV DNA <10 000 copies/mL after 48 weeks off-treatment. At week 96, the SVR rates were 69% and 80% for HBeAg-positive and HBeAg-negative patients with *two-step Pegasys treatment*, compared with 100% (*P* = 0.01) and 100% (*P* = 0.104) in patients with continual entecavir monotherapy, respectively, as shown in Fig. 4a and b. The HBV DNA undetectable rates were 31% and 30% for HBeAg-positive and HBeAg-negative patients with *two-step Pegasys treatment*, compared with 84% (*P* = 0.002) and 92% (*P* = 0.003) in patients with continual entecavir monotherapy, respectively, at week 96.

### ALT levels and hepatitis flares

Patients in Group 1 with high ALT levels (median 723 IU/L; IQR, 596–854) had received entecavir pretreatment for a duration of 19 ± 3.46 days (Table 1). Patients in Group 2 with high ALT levels (median 612 IU/L; IQR, 459–676.5) were under such pretreatment for a period of 20 ± 3.23 days (Table 2). Pegasys was sequentially added once ALT level declined to 200–400 IU/L, (median 298 IU/L; IQR, 230–359) and (median 312 IU/L; IQR, 277–373.5) for Group 1 and Group 2, respectively. Entecavir was discontinued 14 days after initiation of the Pegasys therapy. The ALT levels were below four times of ULN after 4 weeks of Pegasys treatment. Only one patient (1/32) had delay ALT flares (180–220 IU/L) at week 16–24, with low HBV DNA levels (425 IU/mL). At week 96, the rates of ALT normalization were 54% and 70% for HBeAg-positive and HBeAg-negative patients with *two-step Pegasys treatment*, compared with 89% (*P* = 0.022) and 100% (*P* = 0.190) in patients with continual entecavir monotherapy, respectively, as shown in Fig. 4a and b.

### Sustained virological response, relapse and delay response

For HBeAg-positive patients in Group 1, the virological response (VR, HBV DNA <4 logs copies/mL) at the end of treatment was 79% (15/19); sustained VR on 24, 48 and 72 weeks of post-treatment follow-up (after completing 24 weekly injection) were 63.2% (12/19), 61.1% (11/18) and 69.2% (9/13), respectively. The rate of HBeAg seroconversion combined with VR at the end of treatment was 42.1% (8/19), and all eight patients kept the durability to 72 weeks of post-treatment at least. The relapse rates on 24, 48 and 72 weeks of post-treatment follow-up were 26.7% (4/15), 28.6% (4/14) and 23.1% (3/13), respectively; the delay response rates were 2/4, 1/4 and 1/2, respectively. One patient achieved HBeAg loss on 24 weeks of post-treatment follow-up.

For HBeAg-negative patients in Group 2, the VR at the end of treatment was 92.3% (12/13); sustained VR on 24 and 48 weeks of post-treatment follow-up (after completing 48 weekly injection) were 61.5% (8/13) and 80% (8/10), respectively. The relapse rates on 24 and 48 weeks of post-treatment follow-up were 33.3% (4/12) and 11.2% (1/9), respectively. Only one patient did not achieve the VR at the end of treatment and during the follow-up. Thus, analysis of delay response in this group will not be statistically meaningful and will be postponed to future in the next phase.

At week 96, five patients of Group 1 plus Group 2 reporting HBsAg loss reached 21.7% (5/23) by *two-step Pegasys treatment*, and three more patients had low titre of HBsAg (<5 IU/mL) and low HBV DNA levels (<1000 copies/mL) with ALT normalization.

### Efficacy of *two-step Pegasys treatment* in HBeAg-positive *vs* HBeAg-negative CHB

For comparison of the efficacy of *two-step Pegasys treatment* between Group 1 (HBeAg positive) and Group 2 (HBeAg negative), the rates of HBsAg clearance and seroconversion at week 96 were 15.4% (2/13) and 7.7% (1/13) for HBeAg-positive patients; 30% (3/10) and 20% (2/10) for HBeAg-negative patients. The rates of undetectable HBV DNA were 30.8% (4/13) and 30% (3/10, *P* = 0.968); SVR were 69.2% (9/13) and 80% (8/10, *P* = 0.56) for HBeAg-positive and HBeAg-negative patients, respectively, at week 96. No significant difference was observed (Fig. S1).

## Discussion

### Safety

Previous studies showed that interferon treatment was accompanied by an ALT flare in 30–40% of patients. Hepatitis flares are considered to be an indicator of favourable response [15], but they can also lead to hepatic decompensation, especially in patients with underlying cirrhosis. Besides, no interferon treatment was suggested for ALT > 10 × ULN (400 IU/L). In this study, only one (1/32) patient had mild, delay ALT flares (180–220 IU/L) because of immune- or drug-mediated hepatitis at week 16–24. We found no ALT flares because of virus-mediated hepatitis under *two-step Pegasys treatment*, and the treatment provided a safe strategy for chronic hepatitis with acute exacerbation in this study. Nonetheless, we do not recommend Pegasys monotherapy on ALT > 10 × ULN as a routine practice.

### HBV DNA decline is not the predictor of response by *two-step Pegasys treatment*

The rapid decline of HBV DNA levels may be attributed to pretreatment with entecavir (mean 20 days) and hyperimmune response during acute exacerbation of chronic hepatitis B. The HBV DNA levels achieved about a log reduction from baseline and declined to 6.36 log copies/ml (mean level) upon initiation of Pegasys. The entecavir pretreatment was maintained for additional 14 days, forcing the HBV DNA level to decrease further and leading to over 3 log reduction at week 12.

On-treatment monitoring in patients, treated with peginterferon, using HBV DNA and HBsAg levels may optimize individualized prediction of response and can help decide which patients benefit from discontinuing peginterferon [10,16–20]. Serum HBsAg level results from the translation of specific mRNAs generated from cccDNA and potentially from HBV DNA integrated in the host genome. Thus, quantification of HBV DNA and HBsAg provides different information. HBsAg can be an indirect expression of transcriptionally active cccDNA, reflecting the control of the infection achieved by the host’s immune system, while HBV DNA levels reflect active viral replication. The 2009 EASL guidelines stated that ‘in case of a primary nonresponse (failure to achieve a log reduction of HBV DNA from baseline at week 12), interferon treatment should be stopped and replaced by nucleos(t)ides [1].’ Because the first step (entecavir pretreatment) of this study can lead to a log reduction of HBV DNA levels and continues for additional 14 days after initiation of Pegasys, the HBV DNA level is not a suitable factor used to distinguish responders and nonresponders to *two-step Pegasys treatment*. In this case, the HBsAg quantification may be the valuable information by helping to predict the response. HBsAg decline during peginterferon therapy is associated with sustained immune control and can lead to subsequent clearance of HBe and HBsAg. However, this study began from May 2007, and the decline trend of HBsAg levels was monitored every 6 months only.

### Two-step treatment, transient combination

The pretreatment with entecavir is only for a short-term period and discontinues 14 days after initiation of Pegasys. The pretreatment can reduce the HBV DNA levels and inhibit the hyperimmune response before and after initiation of Pegasys. In large randomized phase III studies, which compared lamivudine and peginterferon monotherapy and combination of peginterferon and lamivudine in HBeAg-positive and HBeAg-negative patients, combination of peginterferon and lamivudine resulted in a greater degree of viral load reduction on treatment. However, no significant difference was observed in treatment end points such as sustained viral suppression, HBeAg seroconversion and HBsAg clearance between peginterferon monotherapy and combination therapy. The combination therapy for the whole treatment course could not achieve a better result at the end of treatment and follow-up [21–25]. As the efficacy of long-term follow-up is unknown for the combination therapy (Pegasys plus Entecavir) in chronic hepatitis B with acute exacerbation, the entecavir is designed for induction (12–26 days) until ALT levels decline to 5–10 × ULN and for transient combination (14 days after initiation of Pegasys) afterwards.

### Weight on duration of entecavir pretreatment

Duration of entecavir pretreatment varied among patients (12–26 days variation), depending on the point at which ALT declined to 200–400 IU/mL. The duration (mean) of entecavir pretreatment was 19.32 (min.12–max. 26) days in Group 1; 20.08 (16–26) days in Group 2, considerably shorter than durations of 24, 48, 72 and 96 weeks in the *two-step Pagasys treatment*. Additionally, to ensure the comparable conditions and same number of Pegasys injection given to each patient at same week count of treatment, the beginning of the 2nd stage (Pegasys add-on) was chosen as the starting point in the statistical analysis. The entecavir pretreatment was then viewed as the pre-operating period in this study.

### High efficacy and durability than previous study

In this study, we observed higher rates of HBeAg seroconversion, HBsAg clearance, SVR and durability at week 48 and 96 for both HBeAg-positive and HBeAg-negative patients [20,26–29]. These higher rates were assessed as (i) Involvement of acute exacerbation phase, ALT levels over 10 × ULN and Pegasys initiated on 5–10 × ULN, which differentiated this regimen from other studies of dealing mild elevation of ALT levels (2–5 × ULN) only. (ii) Including pretreatment with entecavir in the method led to lower HBV DNA level. (iii) Treat at the prime time. The time of adding Pegasys is dependent upon ALT levels (5–10 × ULN) after entecavir pretreatment, not a fixed duration. The duration of pretreatment of Group 1 plus Group 2 ranged from 12 to 26 days (mean 19.63 ± 3.34 days), and the HBV DNA levels declined 0.94 log copies/ml when the Pegasys was initiated; the HBV DNA levels became 6.73 and 5.81 log copies/ml for HBeAg-positive and HBeAg-negative patients, respectively. At this moment, with the higher ALT levels (5–10 × ULN) and lower HBV DNA levels (<7 log copies/ml), it is the prime time of initiating Pegasys. (iv) Did genotype B attribute to HBsAg loss? Five patients with HBsAg loss were all genotype B (5/23), and three in the rest (18 patients) of genotype B without HBsAg loss had low titre of HBsAg (<5 IU/mL) and low HBV DNA levels (<1000 copies/mL) at week 96. These three patients have potential of HBsAg loss in the future [20]. However, there was no significant difference (*P* value = 0.128) between genotype B and C.

### No inferior efficacy on HBeAg-negative than HBeAg-positive by two-step Pegasys treatment

HBV DNA suppression to undetectable levels is rarely achieved in HBeAg-negative patients receiving interferon therapy, and these patients frequently relapse after withdrawal of interferon therapy [6,26–29]. However, the efficacy of HBeAg-negative patients (Group 2) treated with the *two-step Pegasys treatment* had noninferior efficacy than HBeAg-positive patients (Group 1) at week 96. Given that the HBeAg-positive patients only received 24 doses of Pegasys, study with extending therapy can further investigate whether better result is achievable.

### Development of the ‘three-step Pegasys treatment’

In the secondary step of *two-step Pegasys treatment*, we must access the on-treatment quantification of HBsAg, HBV DNA levels, tolerability and adverse effects. These factors help to decide whether to stop Pegasys (switch to NA) or to extend length of treatment (48–96 weeks). This step beyond current strategy is called the ‘*three-step Pegasys treatment* for chronic hepatitis B with acute exacerbation’. We did not modify the duration of Pegasys therapy in this study. The extending therapy is favourable to slow responders. Finally, because of the small sample size of *two-step Pegasys treatment*, further investigation is necessary to confirm these trends and phenomena.

In conclusions, while whether the *‘two-step Pegasys treatment’* is the most advantageous strategy of administering Pegasys requires further investigation, it offers a potent alternative, other than the nucleos(t)ides, for treating chronic hepatitis B with acute exacerbation at present. To patients who are suitable for Pegasys treatment and are in chronic hepatitis B with acute exacerbations, if treated at the prime time of offering such protocol, the strategy can achieve high safety and efficacy. Our conclusion remains conservatively because of the small sample size and nonrandomized controlled study.

## Abbreviations:

NA: Nucleoside/nucleotide analogues
Pegasys: Peginterferon α-2a
PEG-INF: pegylated interferon-α
SVR: Sustained virological response
ULN: Upper limit of normal
VR: virological response

## Supporting Information

**Fig. S1** Clinical responses at week 96 for patients with chronic hepatitis B received two-step Pegasys treatment.

**Table S1.** Clinical characteristics of chronichepatitis B patients on ***two-step peginterferonalfa-2a*** treatment and continual Entecavir treatment for 96 weeks study.
