# Supplementary material for: Safety and efficacy of two-step peginterferon α-2a treatment in patients of chronic hepatitis B with acute exacerbation
Source: J Viral Hepat. 2012 Mar;19(3):161–72. doi: 10.1111/j.1365-2893.2011.01469.x (PMC3489065; doi:10.1111/j.1365-2893.2011.01469.x)
Supplement: Supplementary file 1 [file jvh0019-0161-SD1.doc]

**Table S1.** Clinical characteristics of chronic hepatitis B patients on ***two-step peginterferon alfa-2a*** treatment and continual Entecavir treatment for 96 weeks study.

| |  |  | |  | **Two-step Pegasys Treatment (Group 1 + 2)** | **Continual Entecavir Treatment (Group 3 + 4)** | **P-value** | | --- | --- | --- | --- | --- | --- | --- | | **Baseline** | | |  |  |  |  | |  | No. of patients | | | 32 | 59 |  | |  | Age (years) | | | 34.96 ± 5.05 (25.6-43.5) ^ | 35.43 ± 5.70 (24.5-48.6) ^ | 0.6983 | |  | Male sex, n (%) | | | 23 (71.9) | 41 (69.5) | 0.812 | |  | HBeAg positive, n (%) | | | 19 (59.3) | 35 (59.3) |  | |  | Genotype B : C | | | 23 : 9 |  |  | |  | Duration of Entecavir pretreatment (days) | | | 19.63 ± 3.34, (12-26) ^ |  |  | |  | 2-Step Pegasys Treatment: | | |  |  |  | |  |  | 1st step (begin) - ALT (IU/L) | | 658 (555-814) |  | 0.1127 * | |  |  | 2nd step (begin) - ALT (IU/L) | | 312 (256-359) |  | 0.0019 ** | |  | Continual Entecavir Treatment (begin) - ALT (IU/L) | | |  | 415 (284-749) |  | |  | 2-Step Pegasys Treatment: | | |  |  |  | |  |  | 1st step (begin) - HBV DNA (log cps/ml) | | 7.30 ± 1.10 |  | 0.2094 * | |  |  | 2nd step (begin) - HBV DNA (log cps/ml) | | 6.36 ± 1.05 |  | 0.0042 ** | |  | Continual Entecavir Treatment (begin)-HBV DNA (log cps/ml) | | |  | 7.01 ± 1.01 |  | | **Results** | | |  |  |  |  | |  | HBV DNA (log copies/ml) | | |  |  |  | |  |  | | Week 12 | 4.31 ± 0.88 | 4.04 ± 0.80 | 0.1420 | |  |  | | Week 24 φ | 3.04 ± 0.89 | 2.81 ± 0.79 | 0.2023 | |  |  | | Week 48 φφ | 3.26 ± 1.19 | 2.15 ± 0.38 | < 0.0001 | |  |  | | Week 72 | 3.64 ± 1.15 | 2.07 ± 0.22 | < 0.0001 | |  |  | | Week 96 | 3.18 ± 1.09 | 2.03 ± 0.11 | < 0.0001 | |  | HBsAg loss, n/ total (%) ^^ | | |  |  |  | |  |  | | Week 12 | 0/32 ( 0.0) | 0/59 (0.0) |  | |  |  | | Week 24 φ | 3/32 ( 9.4) | 0/59 (0.0) | 0.017 | |  |  | | Week 48 φφ | 5/32 (15.6) | 0/59 (0.0) | 0.002 | |  |  | | Week 72 | 5/31 (16.1) | 0/50 (0.0) | 0.003 | |  |  | | Week 96 | 5/23 (21.7) | 0/31 (0.0) | 0.006 | |  | HBsAg seroconversion, n/ total (%)^^ | | |  |  |  | |  |  | | Week 12 | 0/32 ( 0.0) | 0/59 (0.0) |  | |  |  | | Week 24 φ | 2/32 ( 6.3) | 0/59 (0.0) | 0.052 | |  |  | | Week 48 φφ | 3/32 ( 9.4) | 0/59 (0.0) | 0.017 | |  |  | | Week 72 | 3/31 ( 9.7) | 0/50 (0.0) | 0.025 | |  |  | | Week 96 | 3/23 (13.0) | 0/31 (0.0) | 0.039 | |  |  | |  |  |  |  | |  |  | |  |  |  |  | |  | **Note:** | | Continuous values are expressed as the “mean ± standard deviation” or “median (IQR)”. IQR denotes interquartile range. | | | | |  |  | | ^ mean ± standard deviation (min.-max) |  |  |  | |  |  | | ^^ n/ total : (patient with specified response / total number of patient) at specified week | | |  | |  |  | | * measures at the "begin of Entecavir (1st step)" vs. "begin of continual Entecavir" | | |  | |  |  | | ** measures at the "begin of Pegasys (2nd step)" vs. "begin of continual Entecavir" | | |  | |  |  | | φ end of Pegasys treatment (2nd step) for patients with HBeAg-positive Chronic Hepatitis B | | |  | |  |  | | φφ end of Pegasys treatment (2nd step) for patients with HBeAg-negative Chronic Hepatitis B | | |  | |
| --- | --- | --- | --- | --- | --- | --- | --- | --- | --- | --- | --- | --- | --- | --- | --- | --- | --- | --- | --- | --- | --- | --- | --- | --- | --- | --- | --- | --- | --- | --- | --- | --- | --- | --- | --- | --- | --- | --- | --- | --- | --- | --- | --- | --- | --- | --- | --- | --- | --- | --- | --- | --- | --- | --- | --- | --- | --- | --- | --- | --- | --- | --- | --- | --- | --- | --- | --- | --- | --- | --- | --- | --- | --- | --- | --- | --- | --- | --- | --- | --- | --- | --- | --- | --- | --- | --- | --- | --- | --- | --- | --- | --- | --- | --- | --- | --- | --- | --- | --- | --- | --- | --- | --- | --- | --- | --- | --- | --- | --- | --- | --- | --- | --- | --- | --- | --- | --- | --- | --- | --- | --- | --- | --- | --- | --- | --- | --- | --- | --- | --- | --- | --- | --- | --- | --- | --- | --- | --- | --- | --- | --- | --- | --- | --- | --- | --- | --- | --- | --- | --- | --- | --- | --- | --- | --- | --- | --- | --- | --- | --- | --- | --- | --- | --- | --- | --- | --- | --- | --- | --- | --- | --- | --- | --- | --- | --- | --- | --- | --- | --- | --- | --- | --- | --- | --- | --- | --- | --- | --- | --- | --- | --- | --- | --- | --- | --- | --- | --- | --- | --- | --- | --- | --- | --- | --- | --- | --- | --- | --- | --- | --- | --- | --- | --- | --- | --- | --- | --- | --- | --- | --- | --- | --- | --- | --- | --- | --- | --- | --- | --- | --- | --- | --- | --- | --- | --- | --- | --- | --- | --- | --- | --- | --- | --- | --- | --- | --- | --- | --- | --- | --- | --- | --- | --- | --- | --- | --- | --- | --- | --- | --- | --- | --- | --- | --- | --- | --- | --- | --- | --- | --- | --- | --- | --- | --- | --- | --- | --- | --- | --- | --- | --- | --- | --- | --- | --- | --- | --- | --- | --- | --- | --- | --- | --- | --- | --- | --- | --- | --- | --- | --- | --- | --- | --- | --- | --- | --- | --- |
