# Supplementary figures and images for: Safety and efficacy of two-step peginterferon α-2a treatment in patients of chronic hepatitis B with acute exacerbation
Source: J Viral Hepat. 2012 Mar;19(3):161–72. doi: 10.1111/j.1365-2893.2011.01469.x (PMC3489065; doi:10.1111/j.1365-2893.2011.01469.x)

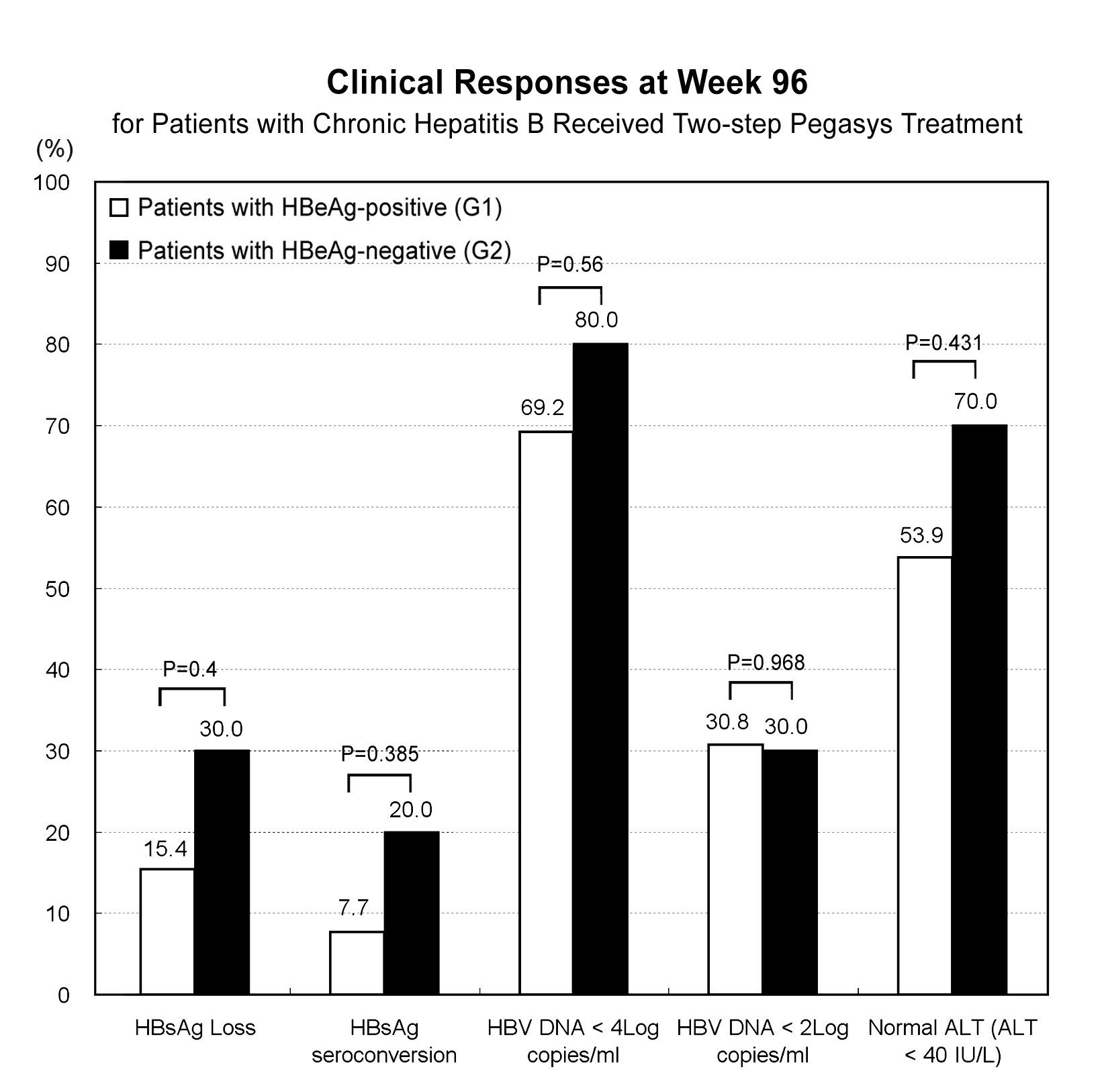

Supplement: Supplementary file 2 [file jvh0019-0161-SD2.tiff]
